# Using Genetic Engineering Techniques to Develop Banana Cultivars With Fusarium Wilt Resistance and Ideal Plant Architecture

**DOI:** 10.3389/fpls.2020.617528

**Published:** 2021-01-13

**Authors:** Xiaoyi Wang, Renbo Yu, Jingyang Li

**Affiliations:** ^1^Key Laboratory of Genetic Improvement of Bananas, Haikou Experimental Station, Chinese Academy of Tropical Agricultural Sciences, Haikou, China; ^2^Key Laboratory of Vegetable Research Center, Tropical Crops Genetic Resources Institute, Chinese Academy of Tropical Agricultural Sciences, Haikou, China

**Keywords:** banana (*Musa* spp.), *Foc* resistance, ideal plant architecture, genetic engineering, genome editing, functional genomics study

## Abstract

Bananas (*Musa* spp.) are an important fruit crop worldwide. The fungus *Fusarium oxysporum* f. sp. *cubense* (*Foc*), which causes Fusarium wilt, is widely regarded as one of the most damaging plant diseases. Fusarium wilt has previously devastated global banana production and continues to do so today. In addition, due to the current use of high-density banana plantations, desirable banana varieties with ideal plant architecture (IPA) possess high lodging resistance, optimum photosynthesis, and efficient water absorption. These properties may help to increase banana production. Genetic engineering is useful for the development of banana varieties with *Foc* resistance and ideal plant architecture due to the sterility of most cultivars. However, the sustained immune response brought about by genetic engineering is always accompanied by yield reductions. To resolve this problem, we should perform functional genetic studies of the *Musa* genome, in conjunction with genome editing experiments, to unravel the molecular mechanisms underlying the immune response and the formation of plant architecture in the banana. Further explorations of the genes associated with *Foc* resistance and ideal architecture might lead to the development of banana varieties with both ideal architecture and pathogen super-resistance. Such varieties will help the banana to remain a staple food worldwide.

## Introduction

Bananas (*Musa* ssp.), which originated in Southeast Asia, are widely cultivated throughout the tropics and sub-tropics, where they represent part of the staple diet and are a vital source of nutrition for over 500 million people (Wang et al., 2019). Due to their widespread popularity, bananas have the largest market share of any fruit worldwide (Langhe et al., 2008). Because bananas are nutritious and starchy, they are considered the fourth most important food crop after rice, wheat, and maize for the alleviation of human starvation in Africa by the United Nations Food and Agriculture Organization (Song et al., 2018). In 2019, worldwide banana production was over 113 million tons; the two largest producers of bananas are India (36.7%) and China (13.8%) (Tzean et al., 2019).

Banana plantations are seriously threatened by various biotic and abiotic stresses, such as diseases, drought, and low temperatures (Michael and Abdou, 2011; Tripathi et al., 2015). At present, the major threat to global banana production is *Foc*, the causative pathogen of Panama disease, which can completely devastate the banana plant. Once established, Fusarium cannot be controlled by any chemical or physical means (Dita et al., 2018). Thus, due to the nutritional and economic importance of bananas, there is a pressing need to develop new cultivars that tolerate *Foc* infection.

In addition, there is a need for bananas with improved plant architecture. This is because, to increase banana production, bananas on small plantations are grown at increasingly high densities (Dash and Rai, 2016). However, dense planting induces competition for light, water, and nutrients (Tian et al., 2019). Thus, the ideal banana architecture includes moderate height, compact leaf angle, and a root system with good hydrotropism; the development of varieties with these characteristics will help to increase banana production (Jiao et al., 2010; Kitomi et al., 2015; Dash and Rai, 2016). However, most widely grown banana varieties with excellent production are *Foc* susceptible, and most *Foc*-resistant banana varieties are poor producers (Heslop-Harrison and Schwarzacher, 2007). To solve this problem, methods should be developed to improve *Foc* resistance as well as plant architecture in bananas, and to balance the need for *Foc* resistance against the need for high production.

The most important cultivated banana varieties worldwide are triploid and originated from the inter- or intra-specific hybridization of two wild diploid species, *Musa acuminate* (A genome) and *M. balbisiana* (B genome) (Wang et al., 2019). Therefore, most banana cultivars are sterile and genetic diversity is narrow, meaning that conventional breeding in this crop is challenging (Heslop-Harrison and Schwarzacher, 2007). Genetic modification may represent a powerful alternative to traditional breeding in bananas, as this method bypasses reproductive barriers and supports the development of improved strains based on excellent cultivars.

Most of the genetic modification methods commonly used in bananas were developed in the 1990s, including protoplast electroporation (Sági et al., 1994), particle bombardment (Sági et al., 1995), and *Agrobacterium*-mediated transformation (May et al., 1995). Embryogenic cell suspensions (ECS) are typical targets of genetic transformation in bananas, and ECS regeneration systems have been established for several banana varieties (Novak et al., 1989; Hu et al., 2013; Dale et al., 2017). To date, several improved transgenic banana varieties with *Foc* resistance and ideal plant architecture have been developed (Table 1). However, the mechanisms underlying *Foc* resistance and ideal plant architecture formation, as well as some critical genes associated with these processes, remain unknown in bananas. The rapid development of genetic engineering techniques, as well as the publication of the banana genome, will greatly facilitate further genetic improvements in bananas (D’Hont et al., 2012; Maxmen, 2019; Wang et al., 2019).

**TABLE 1:** Improved agricultural traits in transgenic bananas (*Musa* spp.).

Trait	Line	Genotype	Explant tissue	Agrobacterium strain	Plasmid	Promoter	Transformed gene	References
*Foc* Race 2 resistance	–	Rasthali (AAB)	ECS	EHA105	pGFP100	pAtUbq3	*MSI-99*	Chakrabarti et al. (2003)
*Foc* Race 1 resistance	VCG 01217	Rasthali (AAB)	Single buds	LBA4404	pROKla	CaMV35S	*GmEg*	Maziah et al. (2007)
	VCG 0124/5	Lady Finger (AAB)	ECS	LBA4404	pPTN254, pPTN261, pPTN396,	pZmUbi	*Bcl-xL, Ced-9, Bcl-2 3′ UTR*	Paul et al. (2011)
	–	Rasthali (AAB)	ECS	EHA105	pCAMBIA1301	pZmUbi	*PhDef1, PhDef2*	Ghag et al. (2012)
	–	Rasthali (AAB)	ECS	EHA105	pCAMBIA1301 + pTZ57R/T	pZmUbi	*VELsen-Int-VELas, FTF1sen-Int-FTF1as*	Ghag et al. (2014)
	–	Rasthali (AAB)	ECS	AGL1	pCAMBIA1305.2	CaMV35S, pZmUbi	*Ace-AMP1 + Ca-pflp*	Sunisha et al. (2020)
TR4 resistance	–	Taijiao (AAA)	Corm slices	Naked gold Particle bombardment + EHA105	pCAMBIA1301	CaMV35S	*Human lysozyme*	Pei et al. (2005)
	–	Pei Chiao (AAA) or Gros Michel (AAA)	MBC	C58C1 or EHA105	pBI121	CaMV35S	*Atfd3, Ca-pflp*	Yip et al. (2011)
	VCG 1213/16	Pisang Nangka (AAB)	CLBs	Particle bombardment	pCambia1304	CaMV35S	*OsTLP*	Mahdavi et al. (2012)
	VCG 01213	Furenzhi (AA)	ECS	EHA105	pCAMBIA1301	CaMV35s	*ThChit42*	Hu et al. (2013)
	-	Williams (AAA)	ECS	EHA105	pCAMBIA1301, pRiAT	CaMV35S	*MaLYK1*, The sense and antisense fragments of *MaLYK1*	Zhang et al. (2019b)
	–	Grand Nain (AAA)	ECS	AGL1	pCAMBIA2200, pPTN261	Nos-P, pZmUbi	*RGA2, Ced-9*	Dale et al. (2017)
	–	Cavendish (AAA)	ECS	EHA105	pCAMBIA1301	pZmUbi	*ERG6, ERG11*	Dou et al. (2019)
Plant height		Gros Michel (AAA)	ECS	EHA105	pYLCRISPR/Cas9P_ubi_-H	OsU6a+OsU3	sgRNA of *MaGA20ox2*	Shao et al. (2019)
Drought tolerance		Rasthali (AAB)	MSCs	EHA105	pCAMBIA1301	pZmUbi	*MaDHN-1*	Shekhawat et al. (2011b)
		Rasthali (AAB)	ECS	EHA105	pCAMBIA1301	pZmUbi	*MaSAP1*	Sreedharan et al. (2012)
		Rasthali (AAB)	ECS	EHA105	pCAMBIA1302	CaMV35S	*MaWRKY71*	Shekhawat and Ganapathi (2013)
		Rasthali (AAB)	ECS	EHA105	pCAMBIA1301	pZmUbi	*MaSNAC1*	Negi et al. (2018)
		Rasthali (AAB)	ECS	EHA105	pCAMBIA1302	CaMV35S	*MaNAC042*	Tak et al. (2017)
		Rasthali (AAB)	ECS	EHA105	pCAMBIA1301	pZmUbi	*MaPIP1;2*	Sreedharan et al. (2013)
		Rasthali (AAB)	ECS	EHA105	pCAMBIA1301	pZmUbi, pMaDHN-1	*MaPIP2;6*	Sreedharan et al. (2015)
		Gongjiao (AA)	Thin cell layers from shoot tips	EHA105	pCAMBIA1302	CaMV35S	*MaPIP2;7*	Xu et al. (2020)
Secondary wall deposition		Rasthali (AAB)	ECS	EHA105	pCAMBIA1301	pZmUbi	*MusaVND1*	Negi et al. (2015)
		Rasthali (AAB)	ECS	EHA105	pCAMBIA1301	pZmUbi	*MusaVND2, MusaVND3*	Negi et al. (2016b)

*Foc, Fusarium oxysporum f. sp. cubense; pZmUbi, the maize polyubiquitin promoter; CLBs, cauliflower-like bodies, induced from the meristemic parts of male inflorescences; MBCs, multiple bud clumps, induced from sucker buds; MSCs, multiple shoot clumps; ECS, embryogenic cell suspensions.*

This study had three main aims: first, to present genetic improvements in *Foc* resistance and plant architecture traits in bananas, with emphasis on those characteristics that are important for banana breeding and those where the underlying molecular mechanisms require further study; second, to show that future efforts to improve *Foc* resistance must also consider the production penalties; and third, to outline the present state of banana genetic transformation technology, and to describe the prospective applications of newly developed genome editing systems in banana breeding.

## Genetic Alterations to Improve *Foc* Tolerance

Fusarium wilt, also known as Panama disease, is one of the most well-known banana disease. This disease, which is caused by the soil-borne fungus *Foc*, destroys the banana plant (Stover and Simmonds, 1987). *Foc* includes four physiologically distinct races: Race 1, Race 2, Race 3, and Race 4; each race has different effects on different host cultivars (Ploetz, 2006; Swarupa et al., 2014). In the 1950s, *Foc* Race 1 devastated the then-dominant cultivar Gros Michel worldwide, leading to the complete replacement of Gros Michel with *Foc* Race 1-resistant Cavendish cultivars (Dita et al., 2018). In the 1990s, another virulent form of *Foc*, tropical race 4 (TR4) seriously damaged Cavendish banana production in Asia (Ploetz, 2006). Since that time, TR4 has invaded Oceania, Australia, and has now reached the Americas (Ploetz, 2006; Maxmen, 2019). Because *Foc* can survive in soil for more than 30 years, it is easily spread by people or equipment carrying contaminated soil (Maxmen, 2019). Once infected with TR4, bananas exhibit discoloration, followed by necrosis in the root and rhizome; due to necrosis, water, and nutrients are not transported, leading to withering and, eventually, the death of the entire plant (Stover and Simmonds, 1987). There is no effective chemical or biological control for TR4 (Dita et al., 2018). As commercially cultivated bananas are sterile and propagated via vegetative suckers, it is not possible to use conventional cross-breeding methods to develop resistance to this fungus. Therefore, global banana production may be seriously devastated by *Foc* in the future unless genetically modified varieties with TR4 resistance are developed (Maxmen, 2019). Several genes that have been used in attempts to develop *Foc* TR4 resistance in bananas are listed in Table 1.

The majority of the transgenic bananas with *Foc* resistance have been developed using antifungal proteins from other crops, such as defensins and ferredoxin-like proteins (Ghag et al., 2012; Sunisha et al., 2020). Transgenic bananas expressing two *defensins* genes from *Petunia* flowers were more resistant to *Foc* Race 1 (Ghag et al., 2012), while those expressing a rice thaumatin-like protein (*tlp*) gene were more resistant to *Foc* Race 4 (Mahdavi et al., 2012). Additionally, potted banana plants transformed with two genes encoding antimicrobial peptides, *antimicrobial peptide* (*Ace-AMP1*) and *ferredoxin-like protein* (*pflp*), exhibited increased resistance to *Foc* Race 1 (vascular discoloration index of 10–20%) as compared to non-transgenic plants (vascular discoloration index of 96%) (Sunisha et al., 2020). *Ace-AMP1* is a non-specific lipid transfer protein (*nsLTPs*) from *Allium cepa* that binds to a signaling receptor to induce a defense response, while the plant *pflp* gene from *Capsicum annum* participates in the activation of the hypersentitive response, initiating systemic acquired resistance (Sunisha et al., 2020). Several other genes have also been overexpressed in bananas to increase Fusarium wilt resistance, such as the soybean β-*1,3-endoglucanase* gene, a rice *chitinase* gene, a sweet pepper *pflp* gene, and synthetic analogs of *magainin* (*MSI-99*), and *human lysozyme (HL)* (Chakrabarti et al., 2003; Pei et al., 2005; Maziah et al., 2007; Yip et al., 2011).

Genes encoding chitinase, which hydrolyzes the main components of the fungal cell wall (chitin and β-glucan), have also been used successfully to inhibit phytopathogenic fungi in various crops (Girhepuje and Shinde, 2011; Chhikara et al., 2012; Das and Rahman, 2012; Nookaraju and Agrawal, 2012). For example, a secreted endochitinase gene from *Trichoderma harzianum (chit42)* improved tolerance to TR4 in transgenic bananas (Hu et al., 2013). Indeed, the levels of *chit42* transcription in transgenic plants are proportional to the level of TR4 resistance (Hu et al., 2013).

Anti-apoptosis genes, which play significant roles in the inhibition of cell death, have been used to increase *Foc* resistance in bananas (Paul et al., 2011; Dale et al., 2017). In glasshouse trials, “Lady Finger” banana plants that had been transformed with various animal genes associated with apoptosis-inhibition [e.g., *B-cell lymphoma-xl* (*Bcl-xL*), *Cell death protein-9* (*Ced-9*), and *Bcl-2* 3′ untranslated region (3′ UTR)] exhibited improved resistance to *Foc* Race 1 (Paul et al., 2011). Similarly, transgenic bananas modified with the *Ced9* anti-apoptosis gene from the nematode *Caenorhabditis elegans* showed improved resistance to TR4 in the field (Dale et al., 2017).

Banana-derived pathogen-resistance genes have also been used to increase disease resistance. For example, a 3 years field trial showed that TR4 resistance was improved in transgenic Cavendish varieties transformed with a resistance gene analog (*RGA2*) from the TR4-resistant wild banana *Musa acuminate malaccensis* (Dale et al., 2017). Similarly, transgenic banana plants overexpressing a banana lysine motif-containing receptor-like kinases 1 (MaLYK1) protein, which plays a role in TR4 resistance by mediating the conserved microbe-associated molecular pattern (MAMP)-activated defense response, had smaller leaf lesions after TR4 treatment than did wild type plants (Zhang et al., 2019b). Indeed, native *Foc*-resistant genes from a number of banana germplasms with *Foc* resistance, such as the double haploid variety Pahang, could be extracted and used to develop transgenic *Foc*-resistant cultivars (Dash and Rai, 2016). Functional genomics studies on these resistant bananas should be performed to reveal the molecular mechanisms underlying the immune response and to explore the important genes associated with *Foc* resistance. Such studies will promote the development of resistant germplasms using transgenic modification techniques.

## Mechanisms Underlying the Defense Response to *Foc* in Plants

The defense mechanisms invoked in response to *Foc* have been investigated in model plants such *Arabidopsis* and tomatoes (Swarupa et al., 2014). Antimicrobial proteins from other crops have been used to improve *Foc* resistance in bananas using transgenic techniques (Swarupa et al., 2014). Due to host specificity, it is necessary to understand the immune mechanisms associated with *Foc* in bananas; this information is necessary to support the rational utilization of the banana’s own resistance-conferring genes in transgenic *Foc-*resistant bananas.

The plant immune system has two layers that sense and defend against pathogens (Swarupa et al., 2014). The first layer is pathogen-triggered immunity (PTI), which is activated by pathogen recognition receptors (PRRs) located on the plant cell surface (Wang W. et al., 2020). In response, adapted pathogens secrete effector proteins to destroy the PTI. Then, the second layer of plant immunity, effector-triggered immunity (ETI), is elicited by the interaction of resistance (R) proteins with pathogen effector proteins (Wang W. et al., 2020). The ETI process is accompanied by the rapid programmed death of the infected cells; this hypersensitive response, which represses pathogen growth and induces the release of toxic pathogenesis-related proteins (PR), is termed systemic acquired resistance (SAR) (Fu and Dong, 2013). Although two banana genes, a PRR gene (*MaLYK1)* and a nucleotide-binding and leucine-rich repeat type R gene (*RGA2*), have been shown to play a role in *Foc* resistance, we still know little about the interaction between bananas and *Foc* (Dale et al., 2017; Zhang et al., 2019b). Using transcriptome profiles, it has been shown that the transcriptional levels of some genes are altered in *Foc*-resistant banana plants during the *Foc* defense response, suggesting that these genes may participate in *Foc* resistance (Li et al., 2012, 2013; Wang et al., 2012; Li W. et al., 2019; Zhang et al., 2019a). For example, several PRR genes, the glucanase gene, cell-wall-strengthening genes, reactive oxygen species (ROS)-scavenging enzyme genes, the non-expression of pathogenesis-related gene 1 (NPR1), and components of the ethylene (ET)/jasmonic acid (JA) biosynthesis and signaling pathway were upregulated in a *Foc*-resistant banana line as compared to a *Foc-*susceptible line in response to TR4 exposure (Li et al., 2012, 2013). Thus, transgenic analyses based on banana genomes should be used to clarify the functions of the identified genes in the *Foc* defense pathway (D’Hont et al., 2012; Wang et al., 2019). Importantly, the publication of the A-genome of a double-haploid *M. acuminate* genotype, DH-Pahang, which is highly resistant to TR4, may provide a valuable research target for studies of the interaction between bananas and *Foc* (D’Hont et al., 2012). Simultaneously, it is vital to investigate the mechanisms that sense *Foc* infection as well as the mechanisms by which R genes resist specific *Foc* effector proteins.

Although the plant immune system plays the primary role in pathogen defense, many environmental factors also heavily influence the interactions between plants and pathogens (Pegg et al., 2019). A dramatic example is the regulation of the plant immune response by light (Gao et al., 2020). As a soil-borne pathogen that infects plants through the roots (in the dark), *Foc* spreads via the vascular system to the aboveground part (in the light), leading to wilting and necrosis (Swarupa et al., 2014). During the infection process, the pathogen experiences a remarkable change in light quality and quantity. That is, the *Foc* infection travels upward along root vascular bundles to the corms and pseudostem of susceptible bananas, transitioning from dark to light during this process. However, this disease progression is nearly inhibited in resistant bananas (Li et al., 2013; Zhang et al., 2019a). It is possible that the light signaling pathway in resistant bananas helps to impede *Foc* progression through the light-exposed, aboveground portion of the plant. Therefore, studies of the effects of light on the *Foc*-banana interaction may help to clarify the immune process in bananas.

## Genetic Alterations to Improve Plant Architecture

Plant architecture, primarily plant height, is an important agronomic trait associated with crop yield (Wang B. et al., 2018). During the first “green revolution” in the middle of the twentieth century, the adoption of semi-dwarf rice, wheat, and maize varieties greatly improved crop production worldwide (Khush, 2001). As banana cultivars are typically more than 2–4 m high, they are vulnerable to lodging during extreme weather events, such as typhoons, and are then not suitable for harvesting (Dash and Rai, 2016). Therefore, dwarf banana cultivars are more suitable for modern intensive planting and fruit harvesting methods (Dash and Rai, 2016).

Semi-dwarf phenotypes of high-yielding varieties were developed during the “Green Revolution” using significant genetic mutations in the gibberellin (GAs) biosynthesis and signaling pathway (Wang et al., 2017). For example, mutations in the *terpene synthase* (*TPS*) gene, the *P450 mono-oxygenases* (*P450s*) gene, the *GA 20-oxidase* (*GA20ox*) gene, and the *GA 3-oxidase* (*GA3ox*) gene impair GA biosynthesis, resulting in the semi-dwarf phenotype (Wang et al., 2017). Recently, the Williams banana mutant, with reduced height compared to its parent, was developed; the reduced height of this mutant was mainly caused by the differential expression of genes involved in GA biosynthesis (Chen et al., 2016). Furthermore, the *GA20ox2* gene has been modified in bananas using the clustered regularly interspaced short palindromic repeat (CRISPR)/CRISPR-associated protein 9 (Cas9) genome editing system to generate a semi-dwarf phenotype of the banana cultivar Gros Michel; this phenotype has lower levels of bioactive GA than the non-transgenic cultivar (Shao et al., 2019). Plant height is also determined by several other hormones, including auxin, brassinosteroids, and strigolactone (Wang B. et al., 2018). For example, the rice *dwarf61* (*d61*), *dwarf14* (*d14*), and *lazy1* mutants exhibit semi-dwarf phenotypes due to inhibited brassinosteroid, strigolactone, and auxin signaling, respectively (Li et al., 2007; Zhao et al., 2014; Wang B. et al., 2018).

Ideally, banana plants should have thick, sturdy stems to improve lodging resistance during adverse weather events, such as typhoons. Increases in secondary wall deposition not only reduce lodging, but also increase plant lignocellulosic biomass (Velasquez Arredondo et al., 2010). Transcription factor NAC plays an important role in the regulation of secondary wall deposition (Negi et al., 2015). In bananas overexpressing *MaVND1*, *MaVND2*, and *MaVND3*, all of which contain an NAC domain, various types of cells transdifferentiated into tracheary element-like cells; in these transgenic bananas, genes associated with xylogenesis were expressed, and ectopic deposition of lignin were observed in various cells (Negi et al., 2015, 2016b). Characterization of the NAC family genes that participate in secondary wall deposition may support the future development of transgenic bananas with improved lodging resistance.

Due to their shallow roots and permanent green canopy, bananas are especially sensitive to water-associated stressors, such as drought and salinity (Sreedharan et al., 2013). In addition, banana roots that are grown under abiotic stress are usually more susceptible to *Foc*, exacerbating decreases in production (Krishna et al., 2015; Pegg et al., 2019). Several genes associated with drought resistance, including those encoding transcription factors (TFs) (e.g., *MaSAP1*, *MaWRKY71*, and *MaNAC*), aquaporins (e.g., *MaPIP1;2*, *MaPIP2;6*, and *MaPIP2;7*), and dehydrin (e.g., *MaDHN-1*) have been successfully incorporated into bananas, and these transgenic lines have developed improved root architecture and increased resistance to drought (Table 1; Shekhawat et al., 2011a; Sreedharan et al., 2012, 2013, 2015; Shekhawat and Ganapathi, 2013; Negi et al., 2015, 2016a,b, 2018; Rustagi et al., 2015; Tak et al., 2017; Xu et al., 2020). These previous studies suggest that banana plants with improved architecture can be developed using genetic strategies.

Recently, proponents of “the second Green Revolution” suggested that the development of crops with IPA would increase crop yield (Jiao et al., 2010). Previous studies in rice and maize have identified several genes that play important roles in increasing grain yield by shaping plant architecture, such as *IPA1*, *TAC1*, and *UPA2* (Jiao et al., 2010; Ku et al., 2011; Dong et al., 2016; Wang B. et al., 2018; Hake and Richardson, 2019; Tian et al., 2019). On banana plantations, cultivars are often planted close together to increase production; as a consequence, banana plants receive insufficient light. Meanwhile, most cultivars have shallow root systems that are susceptible to drought (Dash and Rai, 2016). Therefore, IPA bananas should express a dwarf phenotype, with strong stems, more upright leaves, and a deep root system. Such plants would use light and water more efficiently, have higher lodging resistance, and thus, improved fruit yield.

The banana germplasm contains various valuable genes associated with useful agronomic traits such as height, leaf angle, root distribution, and resistance to abiotic and biotic stresses (Heslop-Harrison and Schwarzacher, 2007). For example, the banana B-genome has long been a target of breeding programs, as these bananas have a high tolerance of abiotic stresses, such as drought (Tripathi et al., 2019). Banana plants are susceptible to drought owing to their shallow roots, large size, and rapid growth (Nansamba et al., 2020). An optimal deep-rooting system may help bananas to resist drought stress by obtaining enough water from deep soil layers (Uga et al., 2011). Thus, studies of the connection between root system architecture and drought tolerance in B-genome bananas might help us to improve drought tolerance in bananas. Conventional gene mapping technologies cannot be used in bananas due to their sterility (Michael and Abdou, 2011). Therefore, genome-wide association studies (GWASs) may help to identify candidate genes associated with desirable traits (Nordborg and Weigel, 2008). GWASs can be used to comprehensively analyze the genomes and phenotypes associated with important agronomic traits (e.g., plant height, leaf angle, and root angle), and to identify the candidate genes underlying these traits (Nordborg and Weigel, 2008).

## The Correlation Between Plant Production and Disease Resistance

The balance between crop production and disease resistance is a major challenge facing modern agriculture (Wang J. et al., 2018). In some cases, the expression of genes associated with increased crop production decreased crop resistance to various pathogens, while the expression of genes improving pathogen resistance decreased crop yield and led to the development of other undesirable agricultural traits during the immune response (Wang J. et al., 2018). Ideal crop varieties should not only have high yields, but also high disease resistance during cultivation (Xu et al., 2017; Wang J. et al., 2018). Recently, several genes have been identified that might improve both crop yield and disease resistance. First, the Ideal Plant Architecture 1 (*IPA1*) gene, which encodes a squamosa promoter binding protein-like 14 (*SPL14*) transcription factor, binds to the promoter of the yield-related genes, increasing plant growth and yield (Wang J. et al., 2018). However, upon pathogen infection, IPA1 is phosphorylated at amino acid Ser163 and instead binds the promoter of WRKY DNA binding protein 45 (*WRKY45*), which enhances immunity to *Magnaporthe oryzae*; at 48 h after *M. oryzae* infection, IPA1 returns to its non-phosphorylated state, re-activating yield-related genes to continue to enhance growth and yield (Wang J. et al., 2018). Second, the Fusarium head blight 7 (*Fhb7*) gene in wheat mediated broad resistance to both Fusarium head blight and crown rot without decreasing wheat yield (Wang H. et al., 2020). Third, transgenic crops with increased resistance to pathogens without decreased fitness have been developed using a TL1-binding transcription factor 1 (*TBF1*) cassette, which contains the immunity-inducible *TBF1* promoter and two pathogen-responsive upstream open reading frames (uORFs) of the *TBF1* gene; this cassette mediates translational control of the expression levels of plants’ own defense genes (Xu et al., 2017).

Similarly, banana breeders must also develop methods by which to control Fusarium wilt while also increasing, or at least not decreasing, yield. During propagation, some somatic clonal variants have been selected based on their *Foc*-resistant characteristics. For example, *Foc*-resistant GCTCV clones, which have excellent agronomic qualities and are *Foc* resistant, were derived from somaconal variants of the Giant Cavendish banana variety (Hwang and Ko, 2004). Some banana varieties, such as FHIA-01 (popularly known as Gold Finger) and Fenza 1, have also been developed for *Foc* resistance, high yield, and unique fruit taste using conventional cross-breeding (Hwang and Ko, 2004; Liu et al., 2012; Smith et al., 2014). However, despite the various breeding programs utilizing beneficial chemical and physical mutations, the number of *Foc*-resistant banana varieties remains limited at present. It is possible that transgenic methods and breeding programs will become important for the development for *Foc*-resistant banana cultivars.

In addition to the previously described genes from rice and wheat that confer both high yield and improved immunity and that can be expressed in bananas using genetic engineering techniques, we should also aim to identify native banana genes that are associated with both excellent immunity and high yield. There are over 1,000 banana cultivars and 180 wild banana species worldwide, representing a wide phenotypic and genomic diversity (Heslop-Harrison and Schwarzacher, 2007). However, due to the genetic sterility of bananas, it is difficult to identify candidate genes associated with desired features via forward genetic methods, such as genetic segregation tests. A re-sequencing dataset generated from five triploid bananas and four diploid bananas contained a total of 18.5 million single-nucleotide polymorphisms (SNPs), 1.4 million small insertions and deletions, and 0.2 million structural variations (Wang et al., 2019). Due to the apparent abundant genetic diversity of polyploid bananas, GWAS is one of the best ways to discover genes and genetic variants related to relevant agronomic traits, as this method identifies statistically important associations between genomic DNA variations in various accessions and target agronomic traits (Zhou and Huang, 2018). In combination, transcriptome analysis, proteome analysis, metabolic profiling, and genomic data can be used to dissect target genes and illustrate the molecular mechanisms underlying the associated agronomic traits (Zhu et al., 2018). Recently, the CRISPR genome-editing system has emerged as a powerful tool for studies of gene function and crop breeding (Feng et al., 2013; Lu et al., 2017). The target specificity of CRISPR is high due to its utilization of short guide RNAs (sgRNAs) (Feng et al., 2013). Therefore, several CRISPR/Cas9-based mutagenesis approaches have been developed for genome-wide mutation screens in mammalian cells and rice using large-scale libraries of synthesized sgRNA (Shalem et al., 2014; Wang et al., 2014; Lu et al., 2017). Using the complete banana genome and the developed ECS methods, whole-genome CRISPR/Cas9 mutant libraries can be constructed for target gene mining, functional gene analysis, and genetic improvement in bananas. In the future, a combination of functional genomic and genetic transformation techniques may lead to the development of improved banana varieties, with both excellent disease resistance and high yield.

## Methods of Genetic Transformation

Banana breeding programs generally aim to improve disease resistance, stress tolerance, yield, and plant architecture (Dash and Rai, 2016). Because conventional breeding is seriously hampered by banana sterility and polyploidy, molecular breeding provides a practical and effective means of genetic improvement (Michael and Abdou, 2011). Two important aspects of banana molecular breeding are efficient regeneration and genetic transformation (May et al., 1995). *In vitro* banana cultures have been developed using various explants, such as shoot tips (Matheka et al., 2019), immature male flowers (Jalil et al., 2003), apical meristems (Novak et al., 1989), corms (May et al., 1995), floral apices (Liu et al., 2016), and ECS (Vuylsteke and Langhe, 1985; Sági et al., 1995; Cote et al., 1996, 2000). Bananas are typically genetically transformed using particle bombardment or *Agrobacterium*-mediated transformation (May et al., 1995; Sági et al., 1995). Previous studies aiming to develop highly efficient, rapid, reproducible, and widely applicable systems for banana genetic transformation and regeneration have used a variety of explant types, banana genotypes, transformation methods, transformation protocols, transformation vectors, and transformation promoters (Table 2). However, even though several varieties of bananas have been genetically transformed and regenerated, the developed methods are variety dependent (Tripathi et al., 2019). Thus, an efficient, stable transformation and regeneration protocol that is applicable to all banana varieties is urgently needed.

**TABLE 2:** Genetic transformations of bananas.

Genotype	Explant tissue	*Agrobacterium* strain	Promoter	Plasmid	Transformed gene	Transformation efficiency	References
SH 3362 (AA), Grand Nain (AAA), Cardaba (ABB), Bodadillo (AA)	Leaf sheaths and corm tissues from the apical meristem	–	–	–	–	–	Novak et al. (1989)
Grand Naine (AAA), Yangambi (AAA), French Plantain (AAB), Mysore (AAB), Silk (AAB), Pelipita (ABB)	ESC from immature male flowers	–	–	–	–	–	Escalant et al. (1994)
Bluggoe (ABB)	Protoplasts	electroporation	CaMV35S, 2 × CaMV35S	pBI221, pBI426, pBI505	*uidA*	Transient expression efficiency is up to 1.8%	Sági et al. (1994)
Bluggoe (ABB), Williams (AAA), Three Hand Planty (AAB)	ESC from proliferating shoot tip buds	Particle bombardment	2 × CaMV35S, pEmu, pUbi	pWRG1515, pBI221, pBI426, pBI505, pAHC27, pEmuGN	*uidA*	25 plants	Sági et al. (1995)
Grand Nain (AAA)	AM, CS	Particle bombardment and LBA4404	OsActin 1	pBI141	*NptII, uidA*	Up to 50%,	May et al. (1995)
Grand Nain (AAA)	ESC from immature male flowers	Particle bombardment	Ubi-1, pBT6.3, CaMV35S	pDHkan, pUGR73, pGEM3Zf^+^, pUC19	ORF of *BBTV DNA-1*, ORF of *BBTV DNA-5*, *nptII, uidA*	11%	Becker et al. (2000)
Rasthali (AAB)	ECS from shoot-tips	EHA105	Gelvin	pVGSUN	*uidA/int*	40 independently transformed plants per 0.5 mL packed cell volume	Ganapathi et al. (2001)
Grand Nain (AAA), Lady Finger (AAB)	ECS From immature male flowers	AGL1, LBA4404	CaMV35S	pCAMBIA1305.1, pART-Test7 (electroporation)	*uidA, GFP*	Up to 65 plants per 50 mg of settled cell volume of ESC	Khanna et al. (2004)
Williams (AAA)	ECS from highly proliferating meristematic tissues	–	–	–	–	–	Chun et al. (2005)
Musa acuminata cv. Mas (AA)	ECS from immature male flowers	EHA105	CaMV35S	pCAMBIA2301	*uidA/int*	490 transgenic plants per 0.5 mL PCV of ECS were obtained	Huang et al. (2007)
Rasthali (AAB)	3 months old sucker	EHA105, EHA101, LBA4404, (sonicated and vacuum infiltered)	CaMV35S	pCAMBIA1301	*uidA*	39.4 ± 0.5%	Subramanyam et al. (2011)
Gonja manjaya (AAB)	ECS from highly proliferative multiple buds	EHA105	CaMV35S	pBI121	*uidA*	50–60 transgenic plants per 0.5 mL settled cell volume	Tripathi et al. (2012)
Grand Naine (AAA)	ECS from immature male flowers	EHA105	pGmHSP17.6-L, pAtHSP18.2	pGW-A	Cre/lox recombination system	702 (41%), 379 (29%) regenerated plants	Chong-Pérez et al. (2012)
Grand Naine (AAA)	ECS from immature male flowers	EHA105	pOsREG-2	pGW-A	Cre/lox recombination system	Up to 76% embryo colonies per plate	Chong-Pérez et al. (2013)
Williams (AAA)	Stable GUS-expressing ECS	EHA105	pZmUbi	pSTARGATE	*gusA*^*INT*^	Up to 100 independent transgenic lines	Dang et al. (2014)
Baxi (AAA), Gongjiao (AA), Red banana (AAA), Rose banana (AA), Xinglongnaijiao (AAB)	Thin cell layers from shoot tips	pCAS04/AGL1 + particle bombardment	Zmubi	pCAS04	*NptII, uidA* without promoter	4.33%-9.81%	Liu et al. (2016)
Gros Michel (AAA)	ECS from the immature male inflorescence	EHA105	OsU6a, OsU3	pYLCRISPR/Cas9Pubi-H	sgRNA of *MaGA20ox2*	152 independent transgenic lines	Shao et al. (2019)
Tai-chiao No.7 (AAA)	Plantlets	EHA105	CaMV35S	pJL89	*MaPDS, MaGSA*	95% efficiency	Tzean et al. (2019)
*Ensete ventricosum* cv. Bedadeti	Multiple buds	EHA105 or LBA4404	CaMV35S	pCAMBIA2300	*GFP*	1.25%	Matheka et al. (2019)
Williams (AAA), Grand Naine (AAA)	ECS from immature male flowers	LBA4404, AGL1	CaMV35S	pMF1	Recombinase-*LBD* gene	Up to 30 transgenic plants	Kleidon et al. (2020)

*ORF, open reading frame; Npt, neomycin phosphotransferase; AM, apical meristem; CS, corm slices; gusA/int, β-glucuronidase gene containing its intron; PCV, packed cell volume.*

During banana genetic transformation, regeneration is generally performed via organogenesis based on meristemic tissue, or via somatic embryogenesis based on ECS (Novak et al., 1989; May et al., 1995). Meristematic tissues, such as shoot tips and floral apices, are widely used as explants for clonal propagation and transformation due to their rapid regeneration times (May et al., 1995; Liu et al., 2016). Although the applicability of this system is limited due to its unavoidable generation of chimeras, it has been shown that two or more cycles of meristem development generated plants with no chimeric tissues (May et al., 1995). To date, the most efficient regeneration and transformation system using longitudinal bud sections as explants was developed by Liu et al. (2016); this system was successfully applied to five banana cultivars: Gongjiao (AA), Red banana (AAA), Rose Banana (AA), Baxi (AAA), and Xinglongnaijiao (ABB) (Liu et al., 2016). In contrast to meristematic tissues, ECSs are an ideal explant for banana genetic transformation, because transgenic plants are derived from a single cell, avoiding the risk of chimeras (Sági et al., 1995). Although the establishment and regeneration of an ECS is time-consuming and inefficient (Novak et al., 1989; Tripathi et al., 2015), ECSs have been successfully obtained from basal leaf sheaths and corm tissues (Novak et al., 1989), highly proliferative meristematic tissue (Chun et al., 2005; Strosse et al., 2006), and immature male flowers (Cote et al., 1996; Dale et al., 2017). Once developed, ECSs can be maintained for 1–1.5 years, although regeneration capacity declines with time (Tripathi et al., 2015). Becker et al. (2000) found that, in Grand Nain bananas, 6 months old embryogenic cells had the greatest regenerative capacity.

## Gene Editing Systems

With the publication of the banana genome and the development of feasible genetic transformation methods, gene editing systems, such as host-induced gene silencing (HIGS) and CRISPR, have become increasingly used to mutate specific genes, leading to the production of mutant plants without having to insert foreign genes (Tripathi et al., 2019; Wang and Chen, 2020).

HIGS has been widely used to develop disease-resistant crops by targeting specific important fungal genes (Huang et al., 2006; Mao et al., 2007). For example, transgenic banana plants carrying small interfering RNA (siRNA) targeting the *Foc* velvet and Fusarium *transcription factor 1* (*ftf1*) genes sustainably resisted *Foc* Race 1 under glasshouse conditions (Ghag et al., 2014). Similarly, transgenic bananas designed to silence the expression of the TR4 ergosterol biosynthesis genes *ERG6* and *ERG11* exhibited significantly improved resistance to TR4 (Dou et al., 2019).

However, the success of HIGS techniques against *Foc* is largely determined by the selection of appropriate target *Foc*-derived genes (Dou et al., 2019). Studies of the *Foc* infection process have identified many key pathogenesis genes in *F. oxysporum* (Li M. et al., 2019). For example, two mitogen-activated protein kinase genes (MAPKs) in *F. oxysporum*, *Fmk1* and *Mpk1*, are associated with cell wall integrity and virulence; the expression of these genes affected the ability of *Foc* to recognize and attach to tomato roots (Segorbe et al., 2017). During *F. oxysporum* invasion and colonization, the sucrose non-fermenting1 (*SNF1*) protein kinase gene regulates the expression of cell wall degrading enzymes (CWDEs) and virulence of *F. oxysporum* (Islam et al., 2017). After infection, a number of virulent *F. oxysporum* toxins lead to the development of disease symptoms in the host; these toxins include effector proteins, which are encoded by the secreted into xylem (*SIX*) genes (Widinugraheni et al., 2018), and fusaric acid (FA), which is encoded by a cluster of FA biosynthetic genes (Ding et al., 2018). However, almost all of these pathogenic mechanisms have been described in Fusarium strains specialized to hosts other than bananas, such as *Fusarium oxysporum* f. sp. *lycopersici* (Li M. et al., 2019). Because *Foc* is strictly host-specialized, we should aim to investigate banana-specific pathogenic and infectious mechanisms in *Foc*. This work will help us to design better targets for the effective control of Fusarium wilt in bananas.

The recently developed precise gene-editing tool CRISPR may be useful for the highly accurate introduction of beneficial genes into the banana, without the concomitant introduction of undesirable foreign genes. The CRISPR genome-editing system includes two key components: an sgRNA that specifically recognizes the target DNA, and a Cas9 endonuclease that precisely cleaves the target DNA (Feng et al., 2013). The most significant advantages of the CRISPR system are twofold: multiple simultaneous mutations and Cas9-free plants (Wang and Chen, 2020). Cas-free transgenic plants are more acceptable to consumers because no foreign genes have been inserted (Maxmen, 2019). In addition, the prolonged presence of Cas9 in a plant might generate off-target mutations. Traditional PCR-based methods and recent fluorescence-based methods for obtaining Cas9-free transgenic plants are based on genetic segregation in sexually propagated plants (Gao et al., 2016; Yu and Zhao, 2019; Wang and Chen, 2020). However, such methods are not applicable in asexually propagated plants like bananas. To solve this problem, preassembled Cas9 protein-gRNA ribonucleoproteins (RNPs), rather than the plasmids that encode these components, are delivered into asexually propagated plant cells (Woo et al., 2015). These RNPs cleave target sites immediately after transfection, and are then rapidly degraded by endogenous proteases in the cell. In this way, targeted mutagenesis in Cas-free regenerated plants is achieved (Woo et al., 2015). Several groups are attempting to improve TR4 resistance in Cavendish bananas using CRISPR, not only by suppressing the expression of the TR4 susceptible gene, but also by expressing dormant genes conferring TR4 resistance (Dale et al., 2017; Maxmen, 2019). The CRISPR system has also been used to develop a semi-dwarf phenotype of the Gros Michel banana cultivar (Shao et al., 2019).

Virus-induced gene silencing (VIGS) can be used to analyze gene function via the transient silencing of RNA expression; VIGS thus compensates for the time-consuming and inefficient nature of transgenic banana production (Baulcombe, 1999). For example, Tzean et al. (2019) developed a VIGS system based on a banana-infecting cucumber mosaic virus (CMV) to efficiently silence target genes. This system might be useful for gene functional analysis in bananas before stable genetic transformation. In summary, the application of various genome editing systems may lead to banana germplasm innovation and additional functional genomics studies of various agricultural traits.

## Challenges and Future Possibilities for the Development of Banana Cultivars With Fusarium Wilt Resistance and Ideal Plant Architecture

Bananas are the fourth most important crop in developing countries after rice, wheat, and maize; these crops are important for food security (Dash and Rai, 2016). The most destructive threat to banana production is Panama disease, caused by *Foc*. Because Fusarium cannot be controlled by any chemical or physical means once established, sustainable banana production requires the use of *Foc*-resistant varieties (Heslop-Harrison and Schwarzacher, 2007). In addition to disease resistance, banana production can also be improved by the development of bananas with IPA (Khush, 2001; Wang et al., 2017). Banana plants with ideal architecture (e.g., dwarfism, strong stems, more upright leaves, and root systems with excellent hydrotropism) would have improved light and water utilization efficiency, lodging resistance, yield, and disease resistance under the current dense planting schemes (Dash and Rai, 2016; Wang J. et al., 2018). It is extremely difficult to develop new banana varieties by conventional breeding methods, due to the sterility, polyploidy, and parthenocarpy of most banana cultivars (Michael and Abdou, 2011). Genetic modification, which compensates for the lack of traditional breeding opportunities, is an effective way to develop bananas with improved agronomic traits, such as increased disease resistance and yield.

Two major challenges face banana breeders aiming to produce genetically modified banana varieties with *Foc* resistance and no yield penalty. First, genes associated with *Foc* resistance, as well as with other important agronomic traits, must be identified, functionally assessed, and utilized in target breeding programs. For example, we should aim to characterize the genes that play a role in *Foc* resistance and ideal plant architecture, such as the specific receptors of effector proteins like SIX, and the genes involved in abiotic resistance. Second, a highly efficient, stable transformation and regeneration system must be developed. The genetic transformation methods available at present are variety dependent, and the regeneration of complete plants from a single transformed cell is difficult (Tripathi et al., 2019).

Functional genomic studies of the *M. acuminate* and *M. balbisiana* genomes will help to clarify the molecular mechanisms underlying the banana immune response, as well as to determine the correlations between disease resistance and banana yield at the genomic level, and to identify the genes associated with pathogen resistance and ideal plant architecture, which can be used for targeted breeding (D’Hont et al., 2012; Wang et al., 2019). In addition, among the *Musa* germplasms are many cultivars and wild species with various agronomic traits, which represent a valuable gene pool resource for banana improvement (Heslop-Harrison and Schwarzacher, 2007). The rapid increase in our understanding of *Musa* genomics, and the development of associated technologies, may help us to better leverage *Musa* biodiversity in targeted molecular breeding programs. Furthermore, due to the host specificity of *Foc* (Swarupa et al., 2014), it is important to investigate its banana-specific pathogenic and infectious mechanisms. An improved understanding of these mechanisms will help us to design siRNAs targeting specific key pathogenic genes and then to use the HIGS system to develop disease-resistant bananas. Finally, once the molecular mechanisms are better understood, genetic engineering techniques, such as precise genome editing, can be used effectively to develop genetically engineered elite banana cultivars with improved disease resistance, ideal architecture, and increased production.

## Author Contributions

XW, RY, and JL conceived, designed the work, and wrote the manuscript. All authors contributed to the article and approved the submitted version.

## Conflict of Interest

The authors declare that the research was conducted in the absence of any commercial or financial relationships that could be construed as a potential conflict of interest.
